# Unlocking Nature’s Microbial Defenders: Genetic Mechanisms and Potential Against *Monilinia* spp. Pathogens

**DOI:** 10.3390/microorganisms13040818

**Published:** 2025-04-03

**Authors:** Augustina Kolytaitė, Ingrida Mažeikienė, Monika Kurgonaitė, Saulė Raklevičiūtė, Gabija Paškevičiūtė, Birutė Frercks

**Affiliations:** Institute of Horticulture, Lithuanian Research Centre for Agriculture and Forestry, Kaunas Str. 30, 54333 Babtai, Lithuania; augustina.kolytaite@lammc.lt (A.K.); monika.kurgonaite@lammc.lt (M.K.); saule.rakleviciute@stud.lsmu.lt (S.R.); birute.frercks@lammc.lt (B.F.)

**Keywords:** microbial antagonists, European plum, brown rot, biological control, whole-genome sequencing

## Abstract

*Monilinia* spp., which causes brown rot, is one of the most damaging pathogens in stone fruits. Researchers are exploring epiphytic and endophytic microorganisms with the potential to suppress pathogens, control pathogenic microorganisms, and/or promote plant growth. In this study, microorganisms with antagonistic activity against three *Monilinia* species were isolated from plum orchard soil and plum fruits. Antagonism tests in vitro showed strong antagonistic properties of six strains of bacteria and two yeast-like fungi against *M. fructigena*, *M. fructicola*, and *M. laxa*, with growth inhibition from 45.5 to 84.6%. The antagonists were identified and characterized at the genetic level using whole genome sequencing (WGS). Genes involved in antibiotic resistance, virulence, secondary metabolite synthesis, and plant growth promotion were identified and characterized through genome mapping, gene prediction, and annotation. None of the microorganisms studied were predicted to be pathogenic to humans. The results of this study indicate that the bacteria *Bacillus pumilus*, *B. velezensis*, two strains of *Lysinibacillus agricola*, *Pseudomonas chlororaphis* isolated from stone fruit orchard soil, and the yeast-like fungus *Aureobasidium pullulans*, isolated from plums, are promising candidates for the biological control of *Monilinia* spp.

## 1. Introduction

The diversity of plant microbiomes can directly or indirectly provide nutrients, help relieve stress, and resist disease [1,2,3]. Microorganisms can regulate plant growth through nitrogen fixation [4], phosphate-solubilization [5], plant hormones secretion [4,6], or siderophore synthesis [7]. Microorganisms are already used for biological control in agriculture. The soil and plant surface are characterized by an endless number of microorganisms with advantageous activities that support plant health [8,9,10]. The bacteria *Pseudomonas*, *Bacillus*, *Burkholderia*, *Streptomyces*, *Serratia*, and *Stephanobacteria*, as well as fungi from the *Trichoderma* genus, are recognized as suitable for biological control, and their synthesized antimicrobial properties and bioactive compounds have been widely studied [11,12,13]. Secondary metabolites, low-molecular-weight antibiotics, and high-molecular-weight antimicrobial proteins or cell wall enzymes—hydrolases—can act as antimicrobial agents [14,15,16,17].

*Monilinia* spp. can cause brown rot, blossom blight, twig cankers, and fruit rot diseases, and are among the world’s most important dangers to stone fruit [18]. The identification of new biological control agents (BCAs) that effectively inhibit disease development is a major challenge for organic horticultural management [19]. Various biocontrol products based on bacterial or yeast antagonists (*Candida oleophila*, *Aureobasidium pullulans*, *Pantoea agglomerans*) have advanced through the development or commercialization stages, and several are successful [20]. In agricultural research, finding novel native biologically active compounds is relevant [21], but processes take too long when classical microbiology methods are used. Whole genome sequencing (WGS) is a technique that provides deep insights into the biosynthetic potential of selected microbial species. WGS analysis of microorganisms allows us to categorize genes associated with antagonistic and plant growth-promoting properties, understand their molecular and functional mechanisms, and thus reduce resources and time in developing biological products [20,21,22,23,24,25].

The aim of this study was to identify microorganisms isolated from soil and plum fruits and describe their properties at the genetic potential level and their antagonistic abilities to inhibit *Monilinia* spp. pathogens. In summary, we provide essential insights into the eight microbial isolates and their antagonistic and plant growth-promoting (PGP) mechanisms, which will benefit future applications aiming at enhancing the biocontrol of *Monilinia* spp. spread in stone orchards.

## 2. Materials and Methods

### 2.1. Isolation of Microorganisms

Microorganisms were isolated from the surrounding soil and fruits of *Prunus domestica* trees, maintained at the collection site of genetic resources for stone fruit orchards (Lithuanian Research Centre for Agriculture and Forestry, Institute of Horticulture, map for 55.0562814 23.8088851) in 2023. The procedure for isolation is as follows. Six genotypes with different resistances to *Monilinia* spp. were chosen: highly resistant No. 293 (free pollination of Cacanska najbolja), resistant No. 202 (Amitar × Jure), No. 219 (free pollination of Cacanska najbolja), No. 242 (Cacanska najbolja × Jure), susceptible No. 212 (Amitar × Jure), and No. 250 (Harmonija × Jure). Isolate names are assigned by tree number. The soil was collected at three places, with the distance from the trunk being 50 cm and the depth being 20 cm, and processed immediately for microbe isolation. The same trees were chosen for the collection of fruits. Five fruits of plums of approximately the same size and with no visual defects were selected at a similar height (around 150 cm) and sun exposure. The fruits were picked wearing sterile gloves, placed into clean plastic bags, and processed to further steps for microbe isolation. The soil and fruits (flesh with skin) were weighed (5 g for each sample). Additionally, fruit samples were homogenized. After adding 10 mL of NaCl 0.9%, the samples were shaken for 30 min at 200 rpm at room temperature. A total of 20 μL of suspension was spread on Petri dishes with lysogeny broth (LB) with agar media in five replicates and incubated for 7 days at 22 °C. Emerging colonies from these plates were further re-streaked until pure colonies were attained. The microbial stock cultures, containing LB and 30% glycerol, were kept at −80 °C until further analysis.

*M. fructigena* MFS01 was isolated, identified, and described by Kolytaite et al. [26]. The cultures of *M. fructicola* CBS 101512 (USA) and *M. laxa* CBS 489.50 (The Netherlands, Baarn) were obtained from Westerdijk Fungal Biodiversity Institute (The Netherlands, Utrecht) (https://wi.knaw.nl/Collection (accessed on 17 June 2024)).

The collection of isolates was stored in deep-freezing conditions according to the description of the rules of the Garden Plant Genetics and Biotechnology Laboratory in the Cryopreservation laboratory of LAMMC in liquid nitrogen.

### 2.2. Evaluation of Morphological Characteristics of Microorganism Isolates

Visual characteristics of all eight plum microbial isolates were evaluated to assess how groups of cells appear in pure cultures on LB agar plates at 22 °C. After 48 h of incubation, the colony shape, elevation, margin, color, smoothness, opacity, consistency, and overall appearance were assessed.

A rub or string test was performed as an alternative to the Gram stain as a confirmatory test [27] for bacterial isolates. A 3% potassium hydroxide solution was used. A small bacterial scrape was placed on a glass slide and, subsequently, one to two drops of the potassium hydroxide (KOH) solution was carefully pipetted on top of the specimen. The resulting suspension was mixed using a sterile plastic loop. Gram-negative bacteria cell walls undergo lysis with 3% KOH, whereas the cell walls of gram-positive bacteria remain intact. Upon lysis of the cell walls of gram-negative bacteria, the release of intracellular DNA occurs, resulting in a viscous (stringy) mixture. The formation of a string in KOH within 60 s is interpreted as a positive test result and indicates that an isolate is a gram-negative organism [27].

### 2.3. In Vitro Inhibition Assay for Monilinia spp.

The inhibition of pathogens *M. fructigena*, *M. fructicola*, and *M. laxa* was assessed using 88 isolated microbial strains. The mycelium plug from the actively growing zone of a 1-week-old fungus (5 mm in diameter) was placed in the middle of the Petri dish containing potato dextrose agar (PDA). Fresh bacterial or yeast-like fungal isolates were streaked in a square pattern around the mycelium plug. Five replicates were created for every microbial isolate. As a control, a plate containing only the fungus disc was used. The dual culture plates were incubated at 22 °C until the mycelium in the control plates reached the edge. Three sites on each plate were used to measure radial growth inhibition, and were determined as follows:

$$\%I=\frac{\left(C-T\right)}{C}\cdot 100$$

where I is the percent of the inhibition of growth, C is the average radius of the fungus on the control plates (5 replicates), and T is the average radius of the fungus on the plates with the isolated microbes (5 replicates).

Antagonistic microorganisms were selected according to the percentage of growth inhibition > 30%. The test was performed using Fisher’s LSD test, followed by Duncan’s multiple range test to test differences between different antagonists.

### 2.4. Evaluation of Genetic Profiles of Potential Antagonists

Only bacteria showing more than 30% inhibition of *Monilinia* spp. were selected for taxonomic identification. Genomic DNA was extracted from an overnight LB liquid cell suspension of isolated microbial antagonists using DNeasy PowerSoil Pro Kit according to the manufacturer’s instructions (Qiagen, Hilden, Germany). DNA quality and concentration were evaluated using NanoPhotometer™ sUV/Vis Spectrophotometer (Implen GmBH, Munich, Germany). Microbial whole genome sequencing was performed at Novogene (Cambridge, UK) using the NovaSeq X Plus Series (PE150) sequencing platform (1 G raw data per sample). Sequences were assembled, and contigs were obtained using Unicycler software (v0.5.1) [28]. BUSCO analysis was used to assess the completeness of the genome assembly [29]. Species were identified using BLAST and the National Center for Biotechnology Information (NCBI) database [30].

The complete genome sequences (cleaned data) of the isolates have been submitted to GenBank under the accession numbers: SAMN44109744, SAMN44109745, SAMN44109747, SAMN44109746, SAMN44109750, SAMN44109751, SAMN44109752, SAMN44109754. Plant growth-promoting genes were predicted using the Kyoto Encyclopedia of Genes and Genomes (KEGG) database [31]. The web-based tool, Antibiotics and Secondary Metabolites Analysis SHell (antiSMASH 7.0) software was used to predict the biosynthesis gene clusters of secondary metabolites in selected isolates [32]. The Comprehensive Antibiotic Resistance Database (CARD) was used to collect information on the putative antimicrobial resistance genes [33]. A web server PathogenFinder [34] and the virulence factor database (VFDB, [35]) were used to predict bacterial pathogenicity. To evaluate the relationships between predicted gene clusters involved in the synthesis of secondary metabolites in antagonists to *Monilinia* spp., a Sankey diagram was generated using SRplot software on web https://sankeymatic.com/ (accessed on 27 January 2025) [36].

## 3. Results

### 3.1. Morphology of Antagonistic Microorganisms

In total, 88 monocultures were isolated and tested against *Monilinia* spp. in vitro. Eight tested microorganisms were selected as strong antagonists against *M. fructicola*, *M. fructigena*, and *M. laxa*, which were used further in our research. Six bacterial isolates were obtained from the soil of the plum orchard. Two microbial strains were isolated from plum fruits and appeared to be yeast-like fungi.

The bacterial isolates with strong antifungal properties were separated into four distinct morphotypes, as shown in Table 1. The isolates could be divided based on several traits: color, form, margin, opacity, appearance, elevation, consistency, and smoothness. Two creamy circular isolates, 202R2 and 293R14, were grouped into the A morphotype. Orange circular isolate R212R1 and white circular isolate 219R3 were placed separately into two different morphotypes, B and C, respectively. Two creamy irregular isolates, R242R3 and 250R3, were grouped into the D morphotype. Most colonies were circular, but R242R3 and 250R3 were irregular. The margin of isolates was entire or undulate. Both isolates 202R2 and 293R14 were opaque. The R212R1 and 219R3 colonies were opaque as well. Both R242R3 and 250R3 isolates were translucent. Morphotypes B and D had a glistening appearance, while morphotypes A and C did not. The investigated isolates were characterized by flat or convex elevation and had butyrous or mucoid consistency. Isolates R212R, R242R3, and 250R3 were smooth, but 202R2, 293R14, and 219R3 had a coarse surface. All isolates were gram-positive, except isolate R212R1, which appeared to be gram-negative.

Two yeast-like fungi isolates (1)R293V2 and R293V5, were separated into distinct morphotype E. They both were creamy, circular, opaque, flat, butyrous, and smooth. The margin was entire, and the appearance was glistening.

### 3.2. Antagonistic Activity of Microorganisms Against Monilinia spp. In Vitro

**Figure 1:**
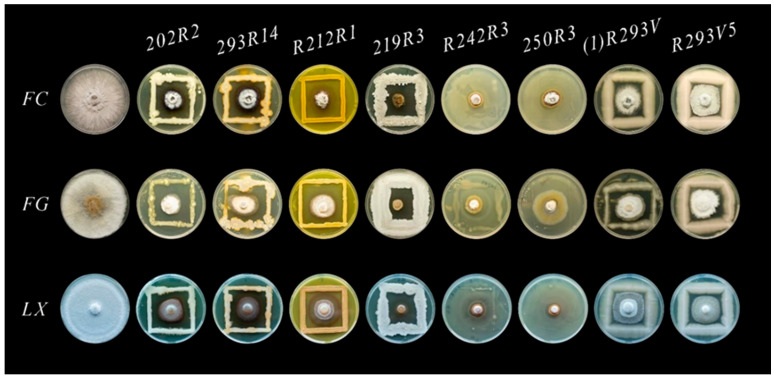
Dual culture antagonism assay of *M. fructicola* (**FC**), *M. fructigena* (**FG**), and *M. laxa* (**LX**) and microbial isolates on PDA, as compared with the control plates (shown on the left).

**Figure 2:**
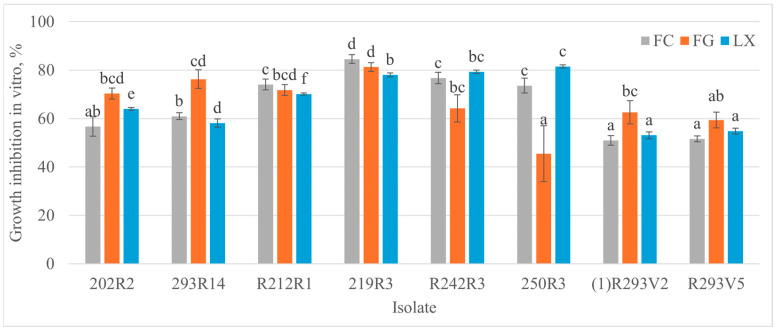
Inhibition of the growth of *M. fructicola* (**FC**), *M. fructigena* (**FG**), and *M. laxa* (**LX**) in vitro on PDA medium as percentages ± SE. The test was performed using Fisher’s LSD and Duncan’s multiple range test. The different letters (a, b, c, d, e and f) above the columns represent significance within the group of antagonists against the same fungal pathogen. Columns, representing average growth inhibition, with the same letters (ab, bcd, bc and cd) are not significantly different (*p* > 0.05). Only columns marked with the same color are comparable for significant differences. LSD 0.05 = 6.77 (for FC); LSD 0.05 = 15,226 (for FG); LSD 0.05 = 2811 (for LX).

The *Monilinia* spp. growth inhibition level varied from 45.5% to 84.6% for eight antagonists (Figure 1 and Figure 2). The highest inhibition percentage rates—81.3% and 84.6% against *M. fructigena* and *M. fructicola*, respectively—were obtained with bacteria 219R3. The highest inhibition rate of 81.5% against *M. laxa* was reached using bacteria 250R3. Both isolates (1)R293V2 and R293V5 with a morphological similarity to yeast-like fungi showed similar antagonism levels for each pathogen. Strong antifungal activity against *M. fructigena* was observed using 202R2 (70.3%) and 293R14 (76.3%) bacteria. The strongest effect on the *M. fructicola*—74.1% and 84.6%—was observed using R212R1 and 219R3 bacteria, respectively. The strongest antagonistic activity against *M. laxa*—79.3% and 81.6%—was reached with R242R3 and 250R3 bacteria, respectively.

### 3.3. Genetic Characteristics of the Isolated Microbial Strains

All morphotypes were identified as bacteria, and the other two isolates proved to be yeast-like fungi upon WGS analysis (Table 2). The morphotype A (202R2 and 293R14) had an identity with the *Bacillus pumilus* reference genome of 95.65% and 95.64%, respectively. The morphotype C (219R3) had 98.87% identity with the *B. velezensis* reference genome. The morphotype D (R242R3, 250R3) was identified as *Lysinibacillus agricola* with identities of 90.65% and 90.71% to the reference genome. Gram-negative morphotype B (R212R1), forming orange colonies, showed the identity of 95.11% with the *Pseudomonas chlororaphis* subsp. *chlororaphis*. Two yeast-like fungi strains, (1)R293V2 and R293V5, were identified as *Aureobasidium pullulans* fungi, with 97% identity to the reference genome.

*B. pumilus* strains 202R2, 293R14, and *B. velezensis* 219R3 genome sizes ranged from ~3.7 to ~3.9 Mb (Table 1). The *P. chlororaphis* isolate had a genome size of ~6.9 Mb, and *L. agricola* isolates’ genome sizes were ~4.9 and ~4.9 Mb, respectively. Eucaryotes *A. pullulans* (isolates (1)R293V2 and R293V5) genome sizes were ~30,364 and 28,396 Mb, respectively. The overall GC content, protein-coding genes’ number, and other genome components (RNAs, repeat regions, and CRISPR) are shown in Table 2. Only *B. pumilus* 202R2, *B. pumilus* 293R14, and *L. agricola* R242R3 were predicted to have 3, 1, and 2 CRISPRs, respectively. The number of coding sequences (CDS) assigned in the NR, Gene Ontology (GO), and KEGG databases of the isolates are shown in Table 2. The CDS differences between isolates of the same species were determined.

### 3.4. Genetic Background for Synthesizing Secondary Metabolites

**Figure 3:**
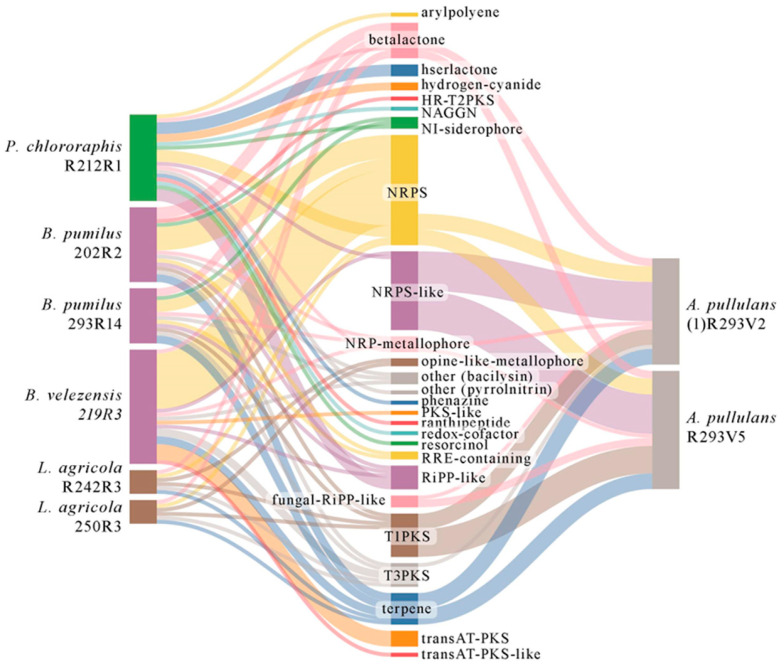
Sankey’s diagram of predicted gene clusters involved in the synthesis of secondary metabolites in the isolated antagonists to *Monilinia* spp., where the different colors represent different gene clusters.

The types of secondary metabolites of each isolate are presented in a Sankey diagram (Figure 3). All analyzed microorganisms were characterized by the synthesis of nonribosomal peptides (NRPSs) and betalactone. NPRS-like enzymes were mostly abundant in fungi *A. pullulans*. They were also detected in bacteria *P. chlororaphis* and *B. velezensis*. A large part (in total 18) of the secondary metabolite types were specific only to bacteria, and one type, the fungal-ribosomally synthesized and post-translationally modified peptides (RiPP)-like gene cluster, was inherent only for fungi. The characteristics of secondary metabolites and comparative analysis with NCBI of the gene clusters are shown in Tables S1–S8. *B. velezensis* 219R3 was predicted to have the highest number of gene clusters responsible for secondary metabolites’ synthesis among selected bacterial isolates. Fungal isolates were identified with an even higher number of gene clusters, but their diversity was greater in bacteria. *A. pullulans* R293V5 was determined to have 26, and *A. pullulans* (1)R293V2 to have 24. *B. velezensis* 219R3 was identified with 21 gene clusters involved in synthesizing secondary metabolites, while *P. chlororaphis* R212R1 had 18, *B. pumilus* 202R2 had 14, and *B. pumilus* 293R14 had 11. *L. agricola* R242R3 and 250R3 were predicted to have five gene clusters involved in synthesizing secondary metabolites, but only this species was characterized by non-ribosomal peptides (NRP)-metallophore synthesis. Both *B. pumilus* isolates and *P. chlororaphis* R212R1 had NI-siderophore clusters, an important factor that helps the plant in nitrogen metabolism.

There were few unique gene clusters specific for isolate *B. pumilus* 202R2 encoded HR-T2PKS; *B. velezensis* 219R3—PKS-like, transAT-PKS and transAT-PKS-like; *P. chlororaphis* R212R1—arylpolyene, hserlactone, hydrogen-cyanide, NAGGN, phenazine, ranthipeptide, redox-cofactor, and resorcinol. After comparison with known secondary metabolite gene clusters, it was predicted that all *Bacillus* spp. isolates might be able to produce bacillibactin and bacilysin. Additionally, *B. pumilus* 293R14 was predicted to synthesize lichenysin and *B. velezensis* 219R3 to synthesize macrolactin H, bacillaene, fengycin, and difficidin. *P. chlororaphis* R212R1 was predicted to synthesize pyrrolnitrin, hydrogen cyanide, and pyocyanine. Both *A. pullulans* isolates were predicted to produce choline and UNII-YC2Q1O94PT. Additionally, it was estimated that *A. pullulans* R293V5 should synthesize chaetoglobosin.

### 3.5. Genes Linked with Plant Growth Promotion (PGP) and Antagonism

Genes related to PGP activity and antagonism in the isolated microbial genomes were screened (Table 3). Phosphate transporters encoded genes involved in phosphate metabolism and tryptophan biosynthesis genes (*trp*) related to IAA synthesis were found in all isolates. Nitrogen metabolism genes were identified for all eight isolates, including *gudB* and *rocG*, which catalyze the reduction of L-glutamate to ammonia; *nasD* and *nasE*—the reduction of nitrite to ammonia; *gltD*—glutamate synthase (NADPH) small chain. The nitrogen-fixation-related genes *nifS* and *nifU* were annotated in *Bacillus* spp. genomes. Additionally, *salA* and *sufU* were identified in *B. velezensis* 219R3. Only *nifU* was found in both *L. agricola* isolates. Siderophore production genes were not found in *P. chlororaphis*, however, in other species they were identified. Hydrolase genes were annotated only for *Bacillus* spp. and fungi *A. pullulans* genomes. Chitinase activity was predicted only for isolates *B. velezensis* 219R3 and *A. pullulans* R293V5. All eight isolated microorganisms were identified as having biofilm synthesis genes as well as some key genes for the synthesis of volatile substances. Homologs of the *ilv* gene for 2,3-butanediol synthesis, or genes *metH* and *ispE* for methanethiol and isoprene synthesis involved in the biocontrol mechanism of antagonistic isolates, were characteristic of all antagonistic microorganisms in this study.

### 3.6. Pathogenicity and Virulence Annotation of Isolated Bacteria

The bacteria pathogenicity was analyzed using PathogenFinder. Both *B. pumilus* isolates matched 16 non-pathogenic families, whereas *B. velezensis* matched 101, *P. chlororaphis* matched 84, *L. agricola* R242R3 matched 26, and *L. agricola* 250R3 matched 28. All *Bacillus* and *Lysinibacillus* spp. isolates did not have any pathogenic families, while *P. chlororaphis* had two of them. All six bacterial isolates were predicted as non-pathogenic microorganisms.

The genome sequences of isolated bacterial isolates were analyzed in the virulence factor database (VFDB), and 30–32 virulence factor genes were identified for *B. pumilus* isolates, 39 for *B. velezensis*, 211 for *P. chlororaphis*, and 29 and 32 for *L. agricola* R242R3 and 250R3 isolates, respectively (Table 4 and Tables S9–S11). These genes were mainly involved in bacterial adhesion, antiphagocytosis, host immune system evasion, iron uptake, regulation, secretion system, and bacterial toxins production (Table 4).

## 4. Discussion

Each class of chemical fungicides against *Monilinia* spp. has a specific target [37,38,39,40], and pathogen resistance develops if used repeatedly [41,42]. Thus, fungicide rotation and combination strategies are essential in treating fungal diseases. A promising agrochemical protection strategy is microbial products and the search for new components for them [43]. The genetic diversity of microorganisms is infinite due to their high adaptability to the environment and the natural interaction between microorganisms. The multicomponent impact of antagonists prevents the pathogen from adapting [44]. This research showed that 8 out of 88 isolated microorganisms had strong antagonistic properties to *Monilinia* spp. pathogens (grow inhibition 45% to 85%) in the microbiota surrounding the plums in the rhizosphere or on the fruit surface (Figure 1 and Figure 2).

According to WGS analysis, three microorganisms were identified as members of the *Bacillus* genus (*B. pumilus* 202R2 and 293R14, *B. velezensis* 219R3) (Table 2). *Bacillus* spp. is widely distributed worldwide and applied in various fields. The isolates R242R3 and 250R3 showed genetic identity with *L. agricola* (access. No. NZ_CP14476 in NCBI)—90.65 and 90.71%, respectively. A cutoff of 97–99% similarity is often used to distinguish species within bacterial taxonomy, but this varies based on the specific group of organisms being studied. This is a novel species of the *Lysinibacillus* genus, first isolated from farmland soil, identified and described in China in 2021 [45]. There are limitations with the reference genome for this species, which means that using it as the standard we cannot yet determine genetic variations. Thus, the one genotype (NZ_CP067341) as a reference genome might not represent the full genetic diversity of a species. We confirmed that both isolates identified in this study have 100% identity among 16s rRNA genes and assigned them to the *L. agricola* species. *Lysinibacillus* shares similarities with *Bacillus* species, but has a unique peptidoglycan composition and fatty acid profile [46]. Morphological characteristics of the isolated R242R3 and 250R3 distinguished them from the others as irregular in form and translucent in opacity (Table 1). Bigger genome sizes with less GC content and fewer gene percentages in the genomes have been identified among these genera in general genome features (Table 2). It is established that *L. agricola* isolates contained 180 and 156 ncRNA regions (R242R3 and 250R3, respectively), while the *Bacillus* genomes contained 16, 14, and 20 ncRNA regions. The ncRNA regions determine a higher complexity of the regulation system and thus better adaptation to stress, increased virulence, and more efficient metabolism in bacteria. *Bacillus* spp. and *Lysinibacillus* spp. have broader applications in agriculture, such as biofertilizers with PGP properties, biopesticides, biofungicides, and bioremediation [46]. *Bacillus* spp. and *Lysinibacillus* spp. are gram-positive and form endospores that can survive in a wide range of environmental conditions and can be formulated into stable dry powders as a product [47]. The gram-negative isolate R212R1 (Table 1) was identified as bacteria *P. chlororaphis* subsp. *chlororaphis* with the homology of 95.11%. *P. chlororaphis* is a plant-associated bacterium with a fast growth rate, wide usage, and various applications, including biocontrol activity, plant growth promotion, and secondary metabolite production [48]. Two fungal isolates—(1)R293V2 and R293V5—were yeast-like fungi *A. pullulans*. This is a black yeast-like fungus of a ubiquitous genus that can survive in diverse environments [49]. *A. pullulans* are found in most phyllosphere and carposphere habitats and possess high antagonistic activity [50].

All isolated antagonists—*B. pumilus*, *B. velezensis*, *L. agricola*, *P. chlororaphis*, and *A. pullulans*—are known as species with antifungal capacity and positive effects on plant growth in agriculture [21,46,51,52,53]. There are several reported strains of *B. pumilus* that showed an antifungal effect on the mycelial growth of *M. fructigena* and *M. fructicola,* by up to ~60% in dual culture assays. *M. laxa* in the same study was inhibited only by up to 30% [21]. In this study, *B. pumilus* isolates showed a similar inhibition effect against *M. fructicola*, but it was stronger against *M. frutigena* and *M. laxa*. There were no reports of *B. velezensis* antagonism against brown rot pathogens, but it is indicated as a broad antagonistic spectrum against various phytopathogenic fungi [51]. In our research, *B. velezensis* 219R3 reached around an 80% antagonism level against all tested *Monilinia* species. *Lysinibacillus* species are known for antagonistic activities against numerous plant pathogens [46,54]. *L. agricola* isolates showed a similar pattern of antagonism against all *Monilinia* spp. pathogens in this study. *P. chlororaphis* is well known for inhibiting various phytopathogens (*Fusarium*, *Colletotrichum*, *Phytophthora*, *Pythium*, *Sclerotinia*, *Magnaporthe oryzae*, *Rhizoctonia*) [52]. *P. chlororaphis* R212R1 inhibition results were ~70% against different *Monilinia* spp. *A. pullulans* washed cells inhibited all three *Monilinia* species on nectarine fruits: *M. laxa* was suppressed completely, and *M. fructicola* and *M. fructigena* growth was reduced by 70–90% [53]. This does not fully align with the results obtained in this research, as *A. pullulans* (1)R293V2 and R293V5 inhibited *M. fructigena*’s growth by ~60%, and *M. fructicola*’s and *M. laxa*’s growth was inhibited by ~50%.

The genetic analysis revealed that identified microorganisms have great genetic potential in synthesizing antifungal compounds like lipopeptides and volatile organic compounds (Figure 3, Tables S1–S8). It was also characterized and confirmed by predicted genes related to PGP activity and antagonism (Table 3). The unique genetic setup of some species in *Bacillus* spp. enables them to produce bacilysin more efficiently than others [54,55]. Bacilysin synthesis is mainly determined by the bac operon and its role in converting prephenate to bacilysin. *B. velezensis* 219R3 was predicted with a high variety of secondary metabolites coding regions. It had 21 clusters, including NRPSs, transAT-PKS, NRP-metallophore, RiPP-like, PKS-like, and terpene. Some of them were identified as locillomycin, surfactin, fengycin, macrolactin H, bacillibactin, bacillaene, bacilysin, butirosin, difficidin, and pliplastin coding metabolites. Studies have reported inhibition of fungal growth using some of these substances through mechanisms such as the disruption of pathogen morphology and physiology [56]. *P. chlororaphis* R212R1 was predicted to synthesize phenazine and resorcinol. Phenazines are pigmented antimicrobial compounds [57]. Both molecules inhibit the growth of various phytopathogens [52]. Additionally, alkyl resorcinol (*dar* gene cluster) produced by *P. chlororaphis* isolate PCL 1606 promotes root colonization by this bacterium and functions antagonistically. They are part of the allelochemical plant-to-plant signaling machinery documented for rice and sorghum [58]. *A. pullulans* (1)R293V2 was predicted to have UNII-YC2Q1O94PT, which encodes melanin [59]. Another isolate, *A. pullulans* R293V5, was predicted with the synthesis of chaetoglobosin A. It was synthesized by *Chaetomium globosum* and has potential antifungal activity [60]. Plant growth-promoting traits might have indirect modes of action against *Monilinia* spp. pathogenesis. All eight isolates were predicted with phosphate, nitrogen metabolism, and IAA production (Table 3). PGP traits are common in the *B. pumilus*, *B. velezensis*, *L. agricola*, *P. chlororaphis*, and *A. pullulans* species [46,52,54,61,62,63] and improve plant metabolism, absorption, and adaptation. All identified isolates had coding genes for biofilm and VOCs (2,3-butanediol, methanethiol, isoprene). In the previous study, the VOCs released by *B. amyloliquefaciens* FZB42 and the formation of biofilms by *B. velezensis* in the plant rhizosphere promoted growth and protected from infectious microbes through systemic resistance [47,64]. Only *B. velezensis* isolate and *A. pullulans* R293V5 were predicted to synthesize chitinase. Additionally, *B. velezensis* and *A. pullulans* R293V5 isolates were predicted with hydrolase activity. In another study, *A. pullulans* antagonistic yeast-like fungi showed enzymatic activity (chitinases, xylanase, urease) [65].

Virulence factors in biofungicides are essential against fungal pathogens. The predicted virulence factors according to VFDB were disclosed in our research (Table 4 and Tables S9–S11). The highest number of virulence factors (211) was found in the *P. chlororaphis* R212R1 genome. The majority (73) were related to adherence, which allows *P. chlororaphis* R212R1 to attach to plant surfaces (roots, leaves, or fungal pathogens). Immune evasion strategies present in three identified *Bacillus* isolates (202R2, 293R14, and R212R1) help bacteria avoid detection, resist immune attacks, and persist within the host. Virulence factors work by breaking down fungal structures, competing for resources, and boosting plant immunity [66]. The eight isolates identified in this study show promise as biocontrol strains for managing *Monilinia*-related infections. *B. velezensis* and *P. chlororaphis* isolates were predicted to have the highest number of secondary metabolites, which might be the reason for the most effective inhibition observed of selected pathogens. For precise application rates and conditions, further studies focusing on *Monilinia* spp. specifically in orchard trials are needed.

## Figures and Tables

**Table 1:** Morphological characteristics of isolated antagonistic bacteria.

Morphotype	A		B	C	D		E	
**Isolate**	202R2	293R14	R212R1	219R3	R242R3	250R3	(1)R293V2	R293V5
**Source**	soil	soil	soil	soil	soil	soil	fruits	fruits
**Color**	creamy		orange	white	creamy		creamy	
**Form**	circular		circular	circular	irregular		circular	
**Margin**	entire			undulate	undulate		entire	
**Opacity**	opaque				translucent		opaque	
**Appearance**	non-glistening		glistening	non-glistening	glistening		glistening	
**Elevation**	flat			convex	flat		flat	
**Consistency**	butyrous			mucoid	butyrous		butyrous	
**Smoothness**	coarse		smooth	coarse	smooth		smooth	
**Gram**	+	+	−	+	+	+	n.a.	

**Table 2:** The general genome features of isolated microbial antagonists.

Characteristics of Microorganisms—Antagonists of *Monilinia* spp.							
*B. pumilus*	*B. pumilus*	*B. velezensis*	*P. chlororaphis* subsp. *chlororaphis*	*L. agricola*	*L. agricola*	*A. pullulans*	*A. pullulans*
**Accession number in NCBI database and isolate strain in current research**							
SAMN 44109744	SAMN 44109745	SAMN 44109747	SAMN 44109746	SAMN 44109750	SAMN 44109751	SAMN 44109752	SAMN 44109754
202R2	293R14	219R3	R212R1	R242R3	250R3	(1)R293V2	R293V5
**Mapping statistics with the reference genome (accession number in NCBI and identity with the reference genome (%)**							
NC_009848		NC_009725	NZ_CP144767	NZ_CP067341	NZ_CP067341	GCF_000721785	
95.65	95.64	98.87	95.11	90.65	90.71	97.06	97.00
**Statistics of sequencing data and annotation** **Genome size (bp)**							
3,733,453	3,721,408	3,976,132	6,995,087	4,901,210	4,923,664	30,364,197	28,396,646
**GC content (%)**							
41.55	41.52	46.36	62.69	36.74	36.66	50.26	50.33
**Gene length (bp)**							
3,312,802	3,313,672	3,520,942	6,125,621	3,922,538	3,964,603	21,770,735	20,714,412
**Genes % in genome**							
88.73	89.04	88.55	87.57	80.03	80.52	71.70	72.95
**Total number of genes**							
3764	3753	3835	6233	4776	4764	8337	5784
**Transfer RNA/Ribosomal RNA (5S, 16S, 23S)/Transfer-messenger RNA**							
70/4/1	70/6/1	83/5/1	61/4/1	80/8/1	75/5/1	158/59/N	156/59—N
**Noncoding RNA**							
16	14	20	62	180	156	78	76
**Repeat region/CRISPR**							
3/3	1/1	N/N	N/N	3/2	N/N	N/N	N/N
**Genes assigned according to NR**							
3425	3654	3489	5672	4346	4335	6886	6256
**Genes assigned according to GO**							
3321	3415	3356	4387	4091	4210	4856	3066
**Genes assigned according to KEGG**							
3078	3002	3321	4128	4005	3845	3798	3088

**Table 3:** Predicted genes related to PGP activity and antagonism in the isolated microbial genomes.

PGP Activities Description	*B. pumilus*		*B. velezensis*	*P. chlororaphis*	*L. agricola*		*A. pullulans*	
	202R2	293R14	219R3	R212R1	R242R3	R250R3	(1)R293V2	R293V5
**Phosphate metabolism**	*pstA*, *pstC*, *pstS*	*pstA*, *pstB*, *pstC*, *pstS*, *iolU*, *mmsA*	*pstA*, *pstB*, *pstC*, *pstS*, *gdh*, *hxlB*, *glpX*, *pfk*, *hxlA*, *kdgK*, *rbsK*	*pstA*, *pstB*, *pstS*, *iolG*, *iolE*, *iolD*, *mmsA*, *iolC*	*pstA*, *pstB*, *pstC*, *pstS*, *mmsA*	*pstA*, *pstB*, *pstC*, *pstS*, *mmsA*	*pstB*, *iolG*, *pstA*, *pstC*, *pstS*, *iolU*	*pstB*, *iolG*, *pstA*, *pstC*, *iolU*
**Nitrogen fixation**	*nifS*, *nifU*	*nifS*, *nifU*	*nifS*, *salA*, *sufU*	-	*nifU*	*nifU*	-	-
**Nitrogen metabolism**	*gltB*, *ltD*, *glnA*	*gltB*, *ltD*, *glnA*	*nasE*, *nasD*, *gudB*, *rocG*, *narG*, *narH*, *narI*, *glnA*, *cah*, *gltB*	*cynS*, *cynT*, *arcC*, *gltB*, *gltD*, *glnA*, *nirB*, *nirD*, *norB*	*cynT*	*cynT*, *glnA*	*ncd2*, *nirB*, *mbtB*, *cah*, *cynT*, *nirD*, *glnA*, *gltB*, *gltD*	*ncd2*, *nirB*, *mbtB*, *cah*, *cynT*, *glnA*, *gltB*
**Siderophore**	*menF*, *entE*, *ntA*	*fhuC*, *enF*	*menF*	-	*fhuC*, *menF*	*fhuC*, *menF*	*fhuC*, *fhuG*, *fhuB*, *fhuD*	*fhuC*, *fhuG*, *huB*, *fhuD*
**IAA production**	*trpA*, *rpB*, *trpC*, *rpD*, *trpE*	*trpA*, *rpB*, *trpC*, *rpD*, *trpE*	*trpA*, *trpB*, *trpC*, *trpD*, *trpE*, *trpF*	*trpA*, *trpB*, *trpC*, *trpD*, *trpE*, *trpF*	*trpA*, *trpB*, *trpE*	*trpA*, *trpB*, *trpE*	*trpA*, *trpB*, *trpD*, *trpE*	*trpA*, *trpE*
**Hydrolase**	*eglS*	*gmuD*, *ganB*	*eglS*	-	-	-	*trpE*, *eglS*, *pqsH*, *pelF*	*eglS*, *pqsH*, *pelF*
**Chitinase activity**	-	-	*ydhD*	-	-	-	-	*sleL*, *ydhD*
**Biofilm**	*trpE*, *flgM*, *fliA*, *csrA*	*trpE*, *flgM*, *fliA*, *csrA*	*tasA*, *bslA*, *bslB*, *trpE*, *flgM*, *csrA*	*crp*, *cpdA*, *trpG*, *trpE*, *flgM*	*trpE*, *fliA*	*trpE*, *fliA*, *csrA*	*tasA*, *bslA*, *bslB*	*tasA*, *bslA*, *bslB*
**2,3-butanediol**	*ilvE*, *ilvA*, *ilvD*, *ilvC*, *ilvB*	*ilvE*, *ilvA*, *ilvD*, *ilvC*, *ilvB*	*ilvE*, *ilvA*, *ilvD*, *ilvC*, *ilvB*	*ilvE*, *ilvA*, *ilvD*, *ilvC*, *ilvB*	*ilvA*, *ilvD*, *ilvC*, *ilvB*	*ilvA*, *ilvD*, *ilvC*, *ilvB*	*ilvY*, *ilvA*, *ilvD*, *ilvC*, *ilvH*	*ilvK*, *ilvE*, *ilvY*, *ilvD*, *ilvC*, *ilvH*, *ilvB*

**Table 4:** Predicted virulence factors in bacterial antagonists according to the virulence factor database (VFDB).

Predicted Virulence Factors	*B. pumilus*		*B. velezensis*	*P. chlororaphis*	*L. agricola*	
	202R2	293R14	219R3	R212R1	R242R3	250R3
Acid resistance	0	0	1	0	0	0
Adherence	2	2	2	73	3	5
Antimicrobial activity	0	0	0	6	0	0
Antiphagocytosis	1	1	2	24	2	1
Biofilm formation	0	0	0	4	0	0
Biosurfactant	0	0	0	1	0	0
Cell surface components	0	0	2	0	0	1
Copper uptake	0	0	0	1	0	0
Efflux pump	0	0	0	1	0	0
Enzyme	1	1	0	1	0	0
Glycosylation system	0	0	0	1	0	1
Immune evasion	10	11	18	8	4	1
Intracellular survival	0	0	0	0	1	1
Invasion	1	1	1	1	0	0
Iron acquisition	5	5	5	0	0	0
Iron uptake	2	2	1	31	7	7
Lipid and fatty acid metabolism	1	1	0	1	1	1
Magnesium uptake	0	0	0	1	0	0
Others	0	0	0	2	0	0
Peptidoglycan modification	1	1	0	0	0	1
Protease	0	0	0	2	0	0
Quorum sensing	0	0	0	5	0	0
Regulation	2	2	2	7	3	3
Secretion system	1	1	1	28	2	2
Serum resistance	0	0	0	1	0	0
Serum and immune evasion	0	0	0	0	1	0
Stress adaptation	0	0	1	4	1	1
Surface protein anchoring	2	2	1	0	1	1
Toxin	1	2	2	8	3	6
**Predicted VF genes**	30	32	39	211	29	32
**Total genes**	3845	3842	4029	6212	4811	5020

## Data Availability

Data are contained within the article or Supplementary Materials.

## Supplementary Materials

The following supporting information can be downloaded at: https://www.mdpi.com/article/10.3390/microorganisms13040818/s1. Tables S1–S8: Predicted gene clusters involved in the synthesis of secondary metabolites. Tables S9–S11: Predicted virulence factors in isolates.
